# Food Neophobia among Adults: Differences in Dietary Patterns, Food Choice Motives, and Food Labels Reading in Poles

**DOI:** 10.3390/nu13051590

**Published:** 2021-05-10

**Authors:** Marzena Jezewska-Zychowicz, Marta Plichta, Małgorzata Ewa Drywień, Jadwiga Hamulka

**Affiliations:** 1 Department of Food Market and Consumer Research, Institute of Human Nutrition Sciences, Warsaw University of Life Sciences (SGGW-WULS), Nowoursynowska 159C, 02-776 Warsaw, Poland; marzena_jezewska_zychowicz@sggw.edu.pl; 2 Department of Human Nutrition, Institute of Human Nutrition Sciences, Warsaw University of Life Sciences (SGGW-WULS), Nowoursynowska 159C, 02-776 Warsaw, Poland; malgorzata_drywien@sggw.edu.pl (M.E.D.); jadwiga_hamulka@sggw.edu.pl (J.H.)

**Keywords:** food neophobia, dietary patterns, food choice, food label reading, adults

## Abstract

Food neophobia (FN) is associated with reduced quality of diet in adults; thus, the understanding of the relationship between FN and food consumption in more depth appears to be a key issue. The aim of the study was to assess the relationship between food neophobia, dietary patterns, food choice motives, and food label reading in the group of adults. Data were collected using the computer-assisted personal interviewing technique (CAPI). A cross-sectional quantitative survey was carried out in November–December 2017 in a sample of 1017 Polish adults. The questionnaire used in the study included the Food Neophobia Scale (FNS), the Beliefs and Eating Habits Questionnaire (KomPAN), and questions regarding food choice motives, reading food labels, and sociodemographic characteristics. The food neophobics were older, had a lower level of education, and had higher BMI compared to others. Compared to others, among the food neophobics, there were more people who often consumed vegetables, fruit, meat, and meat products and who rarely consumed functional and convenience food, sweets, and sweetened beverages. When choosing food, more food neophobics chose healthy and tasteless food products, while more food neophilics chose unhealthy and tasty products. More food neophobics declared not reading price and shelf-life information on food labels compared to the other two groups. Although food neophobia may make adaptation to dietary recommendations difficult, health-promoting features of the diet were observed within the food neophobics. Actions focusing on food choice motives may help even more to limit the effects of food neophobia in adults. Further research is recommended to confirm the observed relationships under different sociocultural conditions.

## 1. Introduction

Food neophobia (FN) is defined as the reluctance to eat or the avoidance of new foods [1]. In the past, it was presumed to be a type of defense mechanism, which prevents the consumption of potentially harmful foods [2]. However, at present, the potential health effects of food neophobia are under debate [3]. It has been shown that food neophobia can not only lead to malnutrition, but also result in limited social functioning and psychological difficulties [4]. Therefore, understanding the relationship between food consumption and food neophobia in more depth appears to be a key issue.

The severity of food neophobia changes throughout the life of an individual and is modulated by various factors [5]. Food neophobia manifests the most in children and is likely to prevent them from experimenting with, and thus experiencing, different types of food [1]. Neophobic children mainly refuse to eat fruit and vegetables rather than other food categories [6]. For example, no correlation has been found so far between food neophobia and the consumption of snacks or starchy staple foods [7]. In general, food neophobia in children is associated with preferences for less healthy foods [8].

The level of neophobia gradually decreases from late childhood until adulthood [9] and slowly starts to rise again with aging, especially in those living alone and having a lower level of education [10,11]. Successful treatment in childhood, for example with the use of cooking-related activities or promotion of flexibility and adjustment in food related situations [12,13], may reduce food neophobia in adulthood, and conversely, if children are not provided with appropriate treatment, food neophobia may follow them into adulthood. Studies on children and adults suggest that food neophobia can not only affect the consumption of healthy foods (i.e., fruit and vegetables) [14], but also reduce the willingness to try healthy food alternatives (e.g., meat substitutes) [15]. Thus, food neophobia is strongly associated with reduced dietary quality in adults [16,17,18]. Neophobic individuals generally consume a lower variety of food [17] and hence may be more exposed to nutritional risks or suffer from specific risks related to a nutrition-deficient diet [19].

Food neophobia can be inherited or can be displaced by environmental influences. Social environments, family, and peers may or may not allow the individuals to display their personality through eating behaviors, and thus follow their tendency toward nutritional neophilia or neophobia [20]. Parents, especially mothers, who play a major role in shaping the eating behaviors of children [21] may transmit to them a tendency toward nutritional neophobia [20]. Therefore, understanding food neophobia in adults is important not only to prevent its health consequences within this group of individuals but also due to its possible impact on people around them.

Reversing the reluctance to accept different foods and ingredients in food neophobics may become critical if their eating behaviors have to be changed. The reasons for changing the eating behaviors may be different, e.g., deteriorating health conditions, preventive health care, or limited access to certain food resources [22,23]. Only a few studies have explored the importance of health in determining the food choice in adults with food neophobia [24,25] and whether they show interest in the information provided by food labels [26]. The results are inconclusive, as when choosing food, the importance of health decreased with increasing food neophobia [24], but also no relationship was shown between the two variables [25]. People with neophobia may look for information on labels to find out if the food product contains ingredients that they usually avoid [27], rather than knowing its nutritional value. Understanding the relationship between food neophobia and the health motive is of great importance when we focus on analyzing the negative impact of food neophobia on food choice and thus on diet. We hypothesize that adults with food neophobia not only consume fewer healthy foods than food neophilics, but also give less importance to health motives when choosing foods and nutritional value when reading food labels than the other group. Thus, the aim of this study was to assess the relationship between food neophobia, dietary patterns, food choice motives, and food labels reading in the group of adult Poles.

## 2. Materials and Methods

### 2.1. Participants and Procedure

A cross-sectional quantitative survey was carried out in November–December 2017. Data were collected using the computer-assisted personal interviewing technique (CAPI) [28]. Participant recruitment and data collection were carried out by a professional market research agency KANTAR, in line with the guidelines of ESOMAR (the European Society for Opinion and Marketing Research) [29]. The study sample was recruited using the stratified-random design from Universal Electronic System of Population Register (PESEL). The details regarding recruitment were previously reported [30]. In brief, participants were selected by systematic drawing based on the classification by region (16 regions) and city size (nine classes). The sample size reflected the demographic structure of the Polish population, in accordance with the public data of the Central Statistical Office in Poland. The adults (over 18 years of age) and those who consented to participate were included in the study. Pregnant and lactating women as well as those with kidney diseases (dialyzed) and neoplastic diseases were excluded. The sample consisted of 1017 adult participants.

### 2.2. Food Neophobia

The level of food neophobia was assessed using the Food Neophobia Scale (FNS) [31]. The translation of the original scale into Polish and its cultural adaptation [32] has been positively verified in the Polish population with good internal consistency [33]. The participants were asked to rate the ten items included in the scale, using a seven-point scale ranging from strongly disagree (1) to strongly agree (7). The FNS includes five positive items (indicating neophilic level) and five negative items (indicating neophobic level). Before the data were analyzed, the scores given for negative items were reversed [34]. Individual FNS scores were calculated by summing the scores of each item of the scale and ranged from 10 to 70. The higher the score, the more neophilic an individual was [31].

### 2.3. Food Choice Motives

The importance of price, taste, and health in food choice was analyzed. For this, a semantic differential question was applied: “Which of the two food products would you choose when making your purchase decision?” Each food product was described using two items, for example items concerning health and taste, items concerning health and price, or items concerning taste and price. The participants chose one of the two products presented in a pair. There were three pairs of products: first pair—healthy and tasteless product (no. 1) and unhealthy and tasty product (no. 2); second pair: healthy and expensive product (no. 1) and unhealthy and cheap product (no. 2); and third pair—tasty and expensive product (no. 1) and tasteless and cheap product (no. 2). The answers given by the participants were rated on a four-point scale as follows: 1—definitely first product; 2—rather first product; 3—rather second product; and 4—definitely second product. In the data analysis, the answers “definitely first product” and “rather first product” were considered as choosing the first product, whereas the answers “definitely second product” and “rather second product” were taken as the choice of the second product.

### 2.4. Reading Food Labels

Food label reading by the participants was assessed by the following question: “Do you pay attention to the information on the labels or packaging of food products?” (answers: “yes” or “no”). Those participants who declared paying attention to such information (answer: “yes”) were required to answer another question: “What information do you read on the labels or packaging of food products?”. For this, the following nine items were presented: price; shelf life; package; product composition; product weight; presence of additives beneficial to health; presence of technological additives; caloric value; and manufacturer. For each item, the participants chose one of the three answers: “yes, very often”, “yes, but sometimes”, or “no”.

### 2.5. Dietary Data

The frequency of consumption of selected food groups was assessed using the Beliefs and Eating Habits Questionnaire (KomPAN) [35]. For this, we regrouped three food groups from KomPAN—cold meats, sausage, and hotdogs—into two groups: (1) cold meats and (2) sausage or hotdogs; vegetables were divided into three groups: (1) green vegetables, (2) red vegetables, and (3) frozen vegetables; fruit were divided into five groups: (1) berries, (2) apples or pears, (3) exotic fruit, (4) frozen fruit, and (5) dried fruit. In addition, we added the following two new food groups: (1) superfood and (2) nuts and seeds. After preliminary analysis, we removed three food groups, namely white bread, butter, and margarine and mixes of butter, because white bread correlated negatively with wholemeal bread and butter correlated negatively with margarine and mixes of butter. These strong correlations would make the interpretation of dietary patterns (DPs) (groups of foods with opposite effects on health) challenging. The participants reported habitual frequency of consumption of each food group within the last three months using the following categories: 1—less than once a month or never; 2—1–3 times a month; 3—once a week; 4—a few times a week; 5—once a day; and 6—a few times a day.

### 2.6. Sociodemographics

The sociodemographic profile of the participants was assessed based on questions related to the following variables: gender; age (in years); place of residence (village, city with less than 100,000 citizens, city with 100,000 citizens or more); education level (primary, vocational, secondary, or higher); and economic situation of household (“we do not have enough money for basic needs”; “we have to be very careful with our daily budget”; “we have enough money for our daily needs, but we need to budget for bigger purchases”; “we have enough money for our needs without particular budgeting”; “we can afford some luxury”). The body mass index (BMI) of the participants was calculated from the self-reported body weight and height. The participants were categorized into four groups based on their BMI, according to the classification of World Health Organization [36]: underweight (BMI < 18.5 kg/m^2^), normal weight (BMI between 18.5 and 24.9 kg/m^2^), overweight (BMI between 25.0 and 29.9 kg/m^2^), and obese (BMI ≥ 30.0 kg/m^2^).

### 2.7. Statistical Analysis

Descriptive statistics were performed. Continuous variables were presented as mean and standard deviation, while categorical variables were presented as sample percentage (%). Factor analysis with the principal component extraction method was applied to derive factors involving reading food labels and DPs, separately. The factors were rotated by an orthogonal (the varimax option) transformation. The number of factors was chosen based on the following criteria: components with an eigenvalue of 1, a scree plot test, and interpretability of the factors. The factorability of the data was confirmed with the Kaiser–Meyer–Olkin (KMO) measure of sampling adequacy and Bartlett’s test of sphericity [37]. For both data sets, the significance of Bartlett’s tests was *p* < 0.001. The KMO value for the factor “reading food labels” was 0.825 and for DPs was 0.898. Loadings equal to 0.50 or higher were used to identify the components of the factors involving reading food labels, while those equal to 0.60 or higher were applied to identify the components of DPs.

The following three factors were identified for food label reading: “Composition and Nutrition Value”, “Price and Shelf Life”, and “Weight, Package, and Manufacturer”. These factors were extracted in a group of participants who declared reading information on food labels or packaging (557 participants). The total variance explained was 63.4%, and the variance explained for each factor was 36.9%, 14.5%, and 11.9%, respectively. The factor-loading matrix for reading food labels is presented in Table 1.

Six dietary patterns were identified as follows: “Functional and Convenience Food,” “Fresh Vegetables and Fruit”, “Meat Products”, “Milk and Dairy Products”, “Sweets and Sweetened Beverages”, and “Cheese”. The total variance explained was 50.2%, and the variance explained for each factor was 20.9%, 9.8%, 7.5%, 4.9%, 3.9%, and 3.2%, respectively. The factor-loading matrix for the DPs identified is presented in Table 2.

Based on tertile distribution, the participants were divided into three groups within both DPs and the factors concerning reading food labels (bottom, middle, and upper tertile). The upper tertile represented the greatest adherence, while the bottom tertile represented the lowest adherence to the DP and the factor concerning reading food labels.

Based on the mean FNS scores and their standard deviation, the participants were divided into three groups as follows: food neophobic (scores lower than 30.8), neutral (scores between 30.8 and 47.0), and food neophilic (scores higher than 47.0). The cut points were at one standard deviation (8.1) from the mean value (38.9). The number of participants in the food neophobic, neutral, and food neophilic groups was 146 (14.4%), 747 (73.4%), and 124 (12.2%), respectively.

The variables were compared using a chi-squared test and one-way analysis of variance ANOVA with post hoc Tukey’s test, with *p* < 0.05 considered statistically significant. Statistical analysis was conducted using Statistica software version 13.3 (StatSoft Inc., Tulsa, OK, USA; StatSoft, Krakow, Poland).

## 3. Results

### 3.1. Sample Characteristics

The characteristics of the participants analyzed in the study are summarized in Table 3. The study sample consisted of a total of 1017 adults, of which 61.3% were female and 38.7% were male. The average age of the participants was 49.3 ± 17.7 years. More than half of the participants lived in cities (51.5%). Those with a secondary level of education accounted for about 43.6% of the sample. Almost two-thirds of the participants (64.8%) declared that they had “enough money for daily needs, but need to budget for bigger purchases”. The mean BMI of the participants was 25.9 ± 4.3 kg/m^2^. The mean level of food neophobia among the participants was determined as 38.9 ± 4.3.

### 3.2. Food Neophobia across Age, Education Level, Economic Situation of Household, and BMI

The characteristics of the sample with respect to the level of food neophobia are presented in Table 3. It was observed that the participants with food neophobia were older than the neutral people and food neophilics. Moreover, they had a lower level of education and higher BMI compared to others. On the other hand, food neophilics were younger, better educated, and had a lower BMI than the other two groups. No significant associations were noted between food neophobia, gender, and place of residence.

### 3.3. Associations between Food Neophobia and Dietary Patterns

The associations between food neophobia and DPs are presented in Table 4. It was observed that among the food neophobics, there were more people who often consumed foods represented by “Fresh Vegetables and Fruit” and “Meat Products” DPs (the upper tertiles) and who rarely consumed foods represented by “Functional and Convenience Food” and “Sweets and Sweetened Beverages” DPs (the bottom tertiles). Among the food neophilics, more people often consumed foods from the DPs “Fresh Vegetables and Fruit” and “Sweets and Sweetened Beverages” (the upper tertiles) and rarely consumed meat and meat products (the bottom tertile of “Meat Products” DP). In the case of neutral participants, there were more people who often consumed foods represented by “Functional and Convenience Food” DP (the upper tertile) and who rarely consumed fresh vegetables and fruit (the bottom tertile of “Fresh Vegetables and Fruit” DP). No association was found between the level of food neophobia and “Milk and Dairy Products” and “Cheese” DPs.

### 3.4. Associations between Food Neophobia and Food Choice Motives

Compared to food neophilics and the neutral participants, more food neophobics chose the “Healthy and Tasteless” food product. On the other hand, more food neophilics chose the “Unhealthy and Tasty” product compared to the neutral group and food neophobics. Moreover, more food neophilics chose the “Tasty and Expensive” product compared to food neophobics and neutral people. In turn, more neutral participants chose the “Tasteless and Cheap” product compared to both food neophobics and food neophilics. No association was noted between the level of food neophobia and choice of “price vs. health” products (Table 5). 

### 3.5. Associations between Food Neophobia and Reading Food Labels

In the study sample, more than half (54.8%, 557 participants) declared that they read food labels. As shown in Table 6, compared to the other two groups, more food neophobics declared that they do not read “Price and Shelf Life” information on food labels (the bottom tertile), while a very few of them read this information (the upper tertile). On the other hand, most of the food neophilics were in the upper tertile of the factor “Price and Shelf Life”, whereas very few of these participants were in the bottom tertile (*p* = 0.007). No differences were noted between food neophobia measured by tertile distribution and reading information related to “Composition and Nutrition Value” and “Weight, Package, and Manufacturer”. However, it was observed that food neophobics were the most diverse in terms of reading information on “Composition and Nutrition Value”, and the group had an equal number of food label readers and nonreaders compared to the other two groups.

## 4. Discussion

The study participants with different levels of food neophobia showed many similarities with the groups that have been studied so far. Food neophobics were shown to be older [9,38,39,40,41], less educated [10,11], and had a higher BMI compared to others [20], while food neophilics were younger, better educated, and had a lower BMI. This is in line with our study in which the food neophobics had, on average, a higher BMI than food neophilics; however, this relationship was more clearly observed in women than in men [20]. We did not find any differences in the level of food neophobia in terms of gender, which was also confirmed in previous studies [39,42], but some authors found that women are more neophobic than men [43,44], while others reported the contrary [10,16]. These differences in obtained results can be explained by the fact that food neophobia can be both inherited and environmentally conditioned [20,34], thus giving rise to cultural differences in the context of food between gender. People living in rural areas have been shown to present a higher level of food neophobia than those living in urban areas [10,45]. However, our results did not confirm this relationship.

The present study showed that people with food neophobia were characterized by a high frequency of consumption of meat and meat products. In general, meat is highly preferred by the Polish population due to various sociocultural conditions. During the end of the 1970s, meat and meat products were inaccessible and considered luxurious goods [46]. The availability of food products, including meat, was regulated by the food rationing system through the introduction of food stamps [47]. Therefore, it can be assumed that older people (over 60 years of age), among whom the highest prevalence of food neophobia (55.0%) was recorded in this study, may currently more often eat meat and meat products due to their negative experiences with the availability of these foods in the earlier years of life. At the same time, younger people more often eliminate meat and meat products from their diet due to their tendency to follow diet trends, including a planetary diet [48]. Their great concern about the problems of environmental protection and ethical reasons related to respect for the life of all living creatures [49,50,51], as well as participation in many healthy eating programs carried out in Poland during the last years, i.e., Wise Nutrition—Healthy Generation Project [52], may explain these limitations in meat consumption in the group of younger people.

On the other hand, a worse economic situation, which is usually associated with a higher level of food neophobia [39,40,53,54], did not limit the consumption of meat and meat products as relatively expensive food [55]. This may mean that food neophobics consume relatively low-quality meat and meat products, which may lead to overweight and obesity and other noncommunicable diseases [56]. Thus, the relationship between a higher level of food neophobia and overweight and obesity shown by other studies is also confirmed by our study [20,53]. In turn, some studies have reported negative correlations between food neophobia and meat consumption [16], which may indicate greater importance of other factors (e.g., demographic or cultural) in determining meat consumption. Less frequent meat consumption by food neophilics may be related to the greater nutritional awareness of younger and educated people [57], who were dominant in this group.

Our results indicated that food neophobics, as often as food neophilics and more often than neutral people, consumed “Fresh Vegetables and Fruit”. The frequent consumption of fruit and vegetables by food neophobic adults is difficult to explain in light of the current knowledge. It is known that neophobic children refuse to eat fruit and vegetables rather than other food categories [6]. In general, it was consistently observed that food neophobia in childhood was associated with less healthy food preferences [8]. Food preferences learned during childhood may persist into adulthood, which has been confirmed in some studies [58,59]. However, our results contradict the findings of other authors and indicate that a higher level of food neophobia is associated with lower consumption of vegetables and fruit [16,17,20,60,61]. On the one hand, the difference in results can be explained by the fact that the declaration of frequent consumption by the food neophobics does not provide information on the amount or variety of consumption of a given product group, which is a significant limitation of our study [62]. On the other hand, the difference may be due to the taste preferences for commonly consumed vegetables and fruit, which in turn translates into eating them more often, as well as in larger quantities. It may suggest that the importance of food neophobia in conditioning eating behaviors in old age is subject to changes. A strong need to maintain health may induce health-promoting behaviors in older people [41], even if they have the desire to avoid certain foods. High consumption of fresh vegetables and fruit allows maintaining a healthy body weight due to their low energy value, while providing a high amount of fiber, vitamins, minerals, and other bioactive compounds that positively influence health [63]. Contrary to a previous study [60], our results showed that people with food neophobia rarely consumed “Sweets and Sweetened Beverages”, which are high-energy products with a low nutritional value [64]. Thus, apart from a high frequency of consumption of meat and its products, food neophobics showed beneficial health behaviors. Other studies showed that food neophobia was associated with decreased consumption or less preference for specific foods, but it had no impact on healthy dietary pattern. Those with a higher level of food neophobia showed lower consumption of fruit and vegetables, but higher consumption of milk, which did not affect the intake of macronutrients and energy, as well as that of sodium, added sugars, and fiber [53]. The observed differences in the eating behaviors of people with food neophobia in relation to previous research results may be related to the ethnic and cultural homogeneity of the Polish population. Thus, the impact of factors that may counterbalance the effect of food neophobia on the eating behaviors of adults should be analyzed in further research.

The present study showed that food neophobics rarely consumed “Functional and Convenience Food”, which is consistent with the results of previous research [65,66] and results related to food neophobia (i.e., reluctance to try new and unknown foods [31], as well as “healthy”, alternative versions of already known dishes and products [15]). The foods categorized under “Functional and Convenience Food” may include those products that are little known to food neophobics, for example, the recently introduced foods on the market. Older people with a higher level of food neophobia are less likely to try new functional foods and avoid their consumption [67,68]. Thus, food neophobia may reduce the consumption of new, including fortified, foods, and thus also foods specially developed for the elderly, which may have a negative impact on their health [11]. In addition to food neophobia, less interest in trying new products among the elderly may result from their more conservative attitude toward food [69].

The level of food neophobia influences the choice of food. Selection of food is a complex process [70], during which individual motives for choice may significantly influence each other. For example, taste may limit the importance of health in food choice [42]. Moreover, price, especially among people with low economic status, may reduce the importance of other motives when deciding about food. In our study, we observed that “Tasteless and Cheap” products were less often chosen by both food neophobics and food neophilics compared to neutral people, which may mean that for the latter, the price of the product is more important than its taste. On the other hand, food neophilics considered taste as more important and health as less important, as most of them chose the “Unhealthy and Tasty” product and the least chose the “Healthy and Tasteless” product. Price did not seem to limit the taste preferences of food neophilics, as most of them chose “the Tasty and Expensive” product, while the least chose the “Tasteless and Cheap” product. On the other hand, most of the food neophobics chose the “Healthy and Tasteless” product, which suggests that health was an important motive for them when choosing food. However, this was not confirmed by previous research [24], which shows that the importance given to factors such as health and natural content decreased with increasing level of food neophobia. On the other hand, the choice of a “Healthy and Tasteless” product may confirm the more frequent consumption of vegetables and fruit and less frequent consumption of sweets in the food neophobic group. Rozin and Falon [71] distinguished three factors underlying the acceptance and rejection of food as follows: (1) expected harmful consequences of consumption; (2) sensory preference; and (3) ideology, which is conditioned by the knowledge about the nature or origin of the substance. In our study, food neophobics seemed to be mostly guided by health rather than taste while preparing food, and rarely consumed sweets and often consumed vegetables and fruit, as well as meat and meat products. Therefore, it can be assumed that the basis for the acceptance and rejection of food by this group was, to a greater extent, the health consequences resulting from food consumption. The exception is the frequent consumption of meat among these participants, which can be explained by referring to their low nutritional knowledge, which does not allow relating the frequent meat consumption with negative health consequences [72]. Moreover, some authors believe that uncertainty about potential health effects of new products and foods is the most important reason for the fear of introducing them into the diet [3], which is confirmed by the rare consumption of “Functional and Convenience Food” declared by food neophobics in this study.

It was surprising that among food neophobics who read food labels, only 36.0% showed interest in information about “Composition and Nutrition Value”. Therefore, it can be assumed that these people obtained such information about food products from other sources (e.g., friends or family) [73]. On the other hand, our results showed that most food neophobics rarely read information on food labels regarding “Price and Shelf Life” compared to other participants. This finding is not surprising, given the importance of health in food selection, and the lower importance of price and flavor. Nevertheless, ignoring the information of use-by date is a serious issue due to the negative health consequences of consuming expired food [74,75]. This lack of interest in shelf life may be due to poor knowledge about the food safety management and general confidence in the food system [76].

### Strengths and Limitations

The strength of our study is the relatively large representative sample of Polish adults. Our study is based on a national population, and the selection of the study group was carried out by professional interviewers. However, our findings are specific to the Polish cultural background and should be used with caution in relation to other populations. The limitations of the study include the large proportion of self-reported data [77] and the cross-sectional design, which did not allow assessing the causality of relationships between the variables. Identification of dietary patterns from the frequency of food consumption is also a limitation due to the overestimation of the consumption of some foods when the frequency of eating is measured [78]. Moreover, the status of food neophobia and the dietary patterns of the participants were not confirmed by experimental methods. However, although the findings should not be generalized to populations with a different cultural background, our study provides an interesting insight into the relationships between food neophobia, food choice motives, eating behaviors, and the habit of reading food labels in adults. This may be of particular importance in light of the current pandemic, fear of limited access to food, and the stress of shopping, especially among the elderly and those living alone.

## 5. Conclusions

Our results showed that, compared with food neophilics, food neophobics comply with dietary recommendations differently, especially in terms of the consumption of meat products and sweets. They more frequently consumed meat products and less frequently consumed sweets. The association between adult food neophobia and limited consumption of vegetables and fruit, which was previously established in children [6], supports the notion that food preferences learned in childhood may change, especially in later adulthood, when health complications develop. The high importance of health accompanied by lower importance of taste in food selection showed by food neophobics may explain the results obtained in this study. Food neophobia should be considered seriously by nutrition counseling professionals, as it may make adaptation to dietary recommendations difficult and thereby also predispose to nutritional deficiencies and noncommunicable chronic diseases. Actions focusing on food choice motives may help to limit the effects of food neophobia in adults. However, further research is recommended to confirm the observed relationships under different sociocultural conditions.

## Figures and Tables

**Table 1:** Factor-loading matrix for reading food labels (*N* = 557).

Variables	Factors		
	Composition and Nutrition Value	Price and Shelf Life	Weight, Package, and Manufacturer
Price	−0.021	0.766 *	0.211
Shelf life	0.135	0.785 *	−0.114
Package	0.129	−0.051	0.807 *
Product weight	0.256	0.421	0.509 *
Manufacturer	0.216	0.103	0.729 *
Product composition	0.764 *	0.170	0.127
Presence of additives beneficial to health	0.833 *	0.047	0.159
Presence of technological additives	0.828 *	0.095	0.101
Energy value	0.714 *	−0.039	0.306
Variance Explained (%)	36.9	14.5	11.9
Total Variance Explained (%)	63.4		
Kaiser’s Measure of Sampling Adequacy	0.825 **		

* Correlation coefficient higher than 0.5; ** *p* < 0.001.

**Table 2:** Factor-loading matrix for the dietary patterns (*N* = 1017).

Variables	Dietary Patterns (Factors)					
	Functional and Convenience Food	Fresh Vegetables and Fruit	Meat Products	Milk and Dairy Products	Sweets and Sweetened Beverages	Cheese
Milk	0.040	−0.090	0.278	0.711 *	0.025	−0.095
Fermented milk beverages (yogurt, kefir, buttermilk)	0.082	0.133	0.011	0.756 *	0.165	0.076
Cottage cheese, homogenized cheese	0.070	0.141	−0.014	0.629 *	−0.036	0.364
Cheese and blue cheese (e.g., Camembert, Brie)	0.076	0.076	−0.062	0.153	0.178	0.732 *
Cold meat (e.g., ham, sirloin)	−0.234	0.155	0.628 *	0.014	0.129	0.138
Sausages and hotdogs	0.129	−0.006	0.731 *	−0.012	0.089	0.005
Green vegetables (e.g., lettuce, cabbage, spinach, broccoli)	0.207	0.604 *	0.111	0.078	0.002	0.085
Red or orange vegetables (e.g., pepper, tomato, carrot)	0.014	0.730 *	0.029	−0.031	0.048	0.170
Apples or pears	−0.024	0.617 *	0.189	0.097	−0.073	−0.063
Exotic fruit (e.g., banana, orange, grapefruit)	0.186	0.618 *	0.068	0.108	0.143	0.045
Frozen vegetables	0.663 *	0.167	−0.145	0.115	−0.026	0.105
Legumes (e.g., bean, pea, lentil, chickpea)	0.615 *	0.253	0.083	0.080	−0.084	−0.033
Berries (e.g., strawberry, raspberry, blueberry, kiwi)	0.633 *	0.262	−0.064	−0.004	−0.000	0.187
Frozen fruit	0.757 *	−0.004	−0.047	0.071	0.021	−0.011
Dried fruit (e.g., apples, apricots, plum, raisins)	0.688 *	0.264	−0.143	0.077	−0.015	0.017
Nuts or seeds	0.605 *	0.362	−0.140	0.090	0.059	0.043
Instant soups or ready-made soups (e.g., tinned, jar, contentrates)	0.678 *	−0.201	0.065	0.038	0.248	0.073
Canned meat	0.658 *	−0.218	0.153	−0.068	0.161	0.050
Superfood (e.g., goji berries, acai berries, vital fiber, milk thistle, psyllium grandmother)	0.635 *	0.037	−0.136	0.091	0.292	−0.100
Sweets (e.g., candy, biscuits, cake, chocolate bars, bars of type “muesli”)	−0.028	0.202	0.060	0.014	0.638 *	0.108
Carbonated or still beverages (e.g., Coca-Cola, Pepsi, Sprite, Fanta, lemonade)	0.146	−0.069	0.264	−0.037	0.736 *	0.002
Variance Explained (%)	20.9	9.8	7.5	4.9	3.9	3.2
Total Variance Explained (%)	50.2					
Kaiser’s Measure of Sampling Adequacy	0.898 **					

* Correlation coefficient higher than 0.6; ** *p* < 0.001.

**Table 3:** Characteristics of the sample according to the level of food neophobia (*N* = 1017).

Variables	Total Sample	Food Neophobia			*p*-Value
		Neophilic	Neutral	Neophobic	
Total *N* (%)	1017 (100.0)	124 (12.2)	747 (73.4)	146 (14.4)	
Gender *N* (%)					
Women	623 (61.3)	75 (12.0)	458 (73.5)	90 (14.3)	0.980 *
Men	394 (38.7)	49 (12.4)	289 (73.4)	56 (14.2)	
Age categories *N* (%)					
Up to 30 years	193 (19.0)	46 (23.8)	137 (71.0)	10 (5.2)	<0.001 *
31–40 years	170 (16.7)	25 (14.7)	134 (78.8)	11 (6.5)	
41–50 years	158 (15.5)	19 (12.0)	124 (78.5)	15 (9.5)	
51–60 years	178 (17.5)	21 (11.8)	133 (74.7)	24 (13.5)	
61–70 years	191 (18.8)	11 (5.8)	132 (69.1)	48 (25.1)	
Over 70 years	127 (12.5)	2 (1.6)	87 (68.5)	38 (29.9)	
Age in years (mean ± SD)	193 (19.0)	39.3 ± 15.2 ^a^	48.9 ± 17.3 ^b^	59.8 ± 16.1 ^c^	<0.001 **
Education level *N* (%)					
Primary	130 (12.8)	5 (3.8)	85 (65.4)	40 (30.8)	<0.001 *
Vocational	304 (29.8)	30 (9.9)	225 (74.0)	49 (16.1)	
Secondary	443 (43.6)	51 (11.5)	345 (77.9)	47 (10.6)	
Higher	140 (13.8)	38 (27.1)	92 (65.8)	10 (7.1)	
Place of residence					
Village	493 (48.5)	56 (11.4)	363 (74.0)	72 (14.6)	0.251 *
City < 100,000 citizens	306 (30.1)	33 (10.8)	224 (73.2)	49 (16.0)	
City ≥ 100,000 citizens	218 (21.4)	35 (16.1)	158 (72.4)	25 (11.5)	
Economic situation of the household *N* (%)					
We do not have enough money for basic needs	50 (4.0)	0 (0.0)	62 (84.2)	3 (15.8)	0.007 *
We have to be very careful with our daily budget	203 (19.9)	11 (12.8)	16 (72.1)	13 (15.1)	
We have enough money for our daily needs, but we need to budget for bigger purchases	659 (64.8)	64 (9.7)	496 (75.3)	99 (15.0)	
We have enough money for our needs without particular budgeting	86 (8.4)	39 (19.2)	31 (70.0)	22 (10.8)	
We can afford some luxury	19 (1.9)	10 (20.0)	142 (62.0)	9 (18.0)	
BMI categories *N* (%)					
Underweight	19 (1.9)	3 (15.8)	13 (68.4)	3 (15.8)	0.005 *
Normal weight	413 (40.6)	67 (16.2)	301 (72.9)	45 (10.9)	
Overweight	429 (42.2)	41 (9.6)	322 (75.1)	66 (15.3)	
Obesity	156 (15.3)	13 (8.3)	111 (71.2)	32 (20.5)	
BMI continuous in kg/m^2^ (mean ± SD)	25.9 ± 4.3	24.8 ± 4.7 ^a^	25.9 ± 4.2 ^b^	26.9 ± 4.6 ^c^	<0.001 **

* Chi-squared test; ** one-way analysis of variance (F); ^a,b,c^ Means differ statistically significantly at *p* < 0.05; *N*—number of participants; %—sample percentage; BMI—body mass index; SD—standard deviation.

**Table 4:** Associations between the level of food neophobia and dietary patterns in the total sample (*N* = 1017).

Dietary Patterns	Food Neophobia			*p*-Value
	Neophilic	Neutral	Neophobic	
	*N* = 124	*N* = 747	*N* = 146	
Functional and Convenience Food *N* (%)				
Bottom tertile	36 (29.0)	229 (30.6)	74 (50.8)	<0.0001
Middle tertile	49 (39.5)	236 (31.6)	54 (36.9)	
Upper tertile	39 (31.5)	282 (37.8)	18 (12.3)	
Fresh Vegetables and Fruit *N* (%)				
Bottom tertile	28 (22.5)	277 (37.1)	34 (23.3)	<0.01
Middle tertile	41 (33.1)	246 (32.9)	52 (35.6)	
Upper tertile	55 (44.4)	224 (30.0)	60 (41.1)	
Meat Products *N* (%)				
Bottom tertile	46 (37.1)	261 (34.9)	32 (21.9)	<0.01
Middle tertile	40 (32.3)	258 (34.6)	41 (28.1)	
Upper tertile	38 (30.6)	228 (30.5)	73 (50.0)	
Milk and Dairy Products *N* (%)				
Bottom tertile	39 (31.5)	243 (32.4)	57 (39.0)	0.630
Middle tertile	43 (34.7)	252 (33.7)	44 (30.2)	
Upper tertile	42 (33.8)	252 (33.7)	45 (30.8)	
Sweets and Sweetened Beverages *N* (%)				
Bottom tertile	30 (24.2)	246 (32.9)	63 (43.2)	<0.001
Middle tertile	32 (25.8)	256 (34.3)	51 (34.9)	
Upper tertile	62 (50.0)	245 (32.8)	32 (21.9)	
Cheese *N* (%)				
Bottom tertile	36 (29.0)	240 (32.1)	63 (43.2)	0.088
Middle tertile	42 (33.9)	255 (34.1)	42 (28.7)	
Upper tertile	46 (37.1)	252 (33.8)	41 (28.1)	

*N*—number of participants; %—sample percentage.

**Table 5:** Associations between food neophobia and food choice in the total sample (*N* = 1017).

Variables	Total Sample	Choice between Two Products:		*p*-Value	Choice between Two Products:		*p*-Value	Choice between Two Products:		*p*-Value
		Healthy and Tasteless Product	Unhealthy and Tasty Product		Healthy and Expensive Product	Unhealthy and Cheap Product		Tasty and Expensive Product	Tasteless and Cheap Product	
	*N* = 1017	*N* = 538	*N* = 479		*N* = 814	*N* = 203		*N* = 866	*N* = 151	
Food Neophobia *N* (%)										
Neophilic	124 (12.2)	49 (39.5)	75 (60.5)	0.004	101 (81.5)	23 (18.4)	0.222	116 (93.5)	8 (6.5)	<0.001
Neutral	747 (73.4)	404 (54.1)	343 (45.9)		589 (78.8)	158 (21.2)		616 (82.5)	131 (17.5)	
Neophobic	146 (14.4)	85 (58.2)	61 (41.8)		124 (84.9)	22 (15.1)		134 (91.8)	12 (8.2)	

*N*—number of participants; %—sample percentage.

**Table 6:** Associations between food neophobia and reading food labels (*N* = 557).

Information on the Labels/Packaging	Food Neophobia			*p*-Value
	Neophilic	Neutral	Neophobic	
	*N* = 75 (100.0)	*N* = 407 (100.0)	*N* = 75 (100.0)	
Composition and Nutrition Value *N* (%)				
Bottom tertile	28 (37.3)	125 (30.7)	33 (44.0)	0.051
Middle tertile	23 (30.7)	149 (36.6)	15 (20.0)	
Upper tertile	24 (32.0)	133 (32.7)	27 (36.0)	
Price and Shelf Life *N* (%)				
Bottom tertile	21 (28.0)	131 (32.2)	39 (52.0)	0.007
Middle tertile	23 (30.7)	139 (34.2)	19 (25.3)	
Upper tertile	31 (41.3)	137 (33.6)	17 (22.7)	
Weight, Package, and Manufacturer *N* (%)				
Bottom tertile	27 (36.0)	136 (33.4)	23 (30.7)	0.732
Middle tertile	20 (26.7)	139 (34.2)	27 (36.0)	
Upper tertile	28 (37.3)	132 (32.4)	25 (33.3)	

*N*—number of participants; %—sample percentage.

## Data Availability

The data presented in this study are available on request from the corresponding author.
